# The complete mitochondrial genome of a new geographical population of freshwater fish *Macropodus hongkongensis* (Freyhof & Herder, 2002)

**DOI:** 10.1080/23802359.2023.2278819

**Published:** 2023-11-23

**Authors:** Qingfeng Zhang, Fangyuan Li, Gaojun Li, Yang Dong, Ji Wang, Dikai Chen, Zhixin Shen

**Affiliations:** a Research Institute of Freshwater Fisheries, Hainan Academy of Ocean and Fisheries Sciences, Haikou, China; b Research Center for Freshwater Bioresource and Eco-environment Protection in Hainan Province, Haikou, China

**Keywords:** *Macropodus hongkongensis*, mitochondrial genome, phylogenetic analysis, new geographical population

## Abstract

*Macropodus hongkongensis* (Freyhof & Herder, 2002), is sparsely distributed in Hong Kong and Guangdong provinces. Recently, a new geographical population of *M. hongkongensis* was discovered in the Wanquan River in the Hainan province. Therefore, this study focused on sequencing the complete mitochondrial genome of the new geographical population of *Macropodus hongkongensis* from the Wanquan River. The circular mtDNA molecule was 16,492 bp in size, and the overall base compositions were A (30.30%), C (24.90%), T (29.80%), and G (15.00%), with a slight bias toward A + T. The complete mitogenome encoded 13 protein-coding genes (PCGs), 22 tRNA genes, 2 rRNA genes, and a control region. Phylogenetic analysis indicated that *M. hongkongensis* of the Hainan Wanquan River was most closely related to the *M. hongkongensis* of the Gongdong population. These results provide useful genetic information for species identification and phylogenetic studies of *Macropodus* species.

## Introduction

*Macropodus hongkongensis*, commonly known as the Hong Kong paradise fish, belonging to the genus *Macropodus* of the family Belontiida and is sparsely distributed in Hong Kong and Guangdong provinces (Chan et al. 2008; Wang et al. 2009). Recently, a new geographical population of *M. hongkongensis* was discovered in the Wanquan River in the Hainan province. The known geographical distribution area of *M. hongkongensis* in Hainan is limited, and its habitat consists of small streams in the intermountain thickets (Shen et al. 2021). In this study, the complete mitogenome of *M. hongkongensis* of the Wanquan River, a new geographical location, was determined and its phylogenetic relationship was analyzed.

## Materials and methods

**Figure 1.**
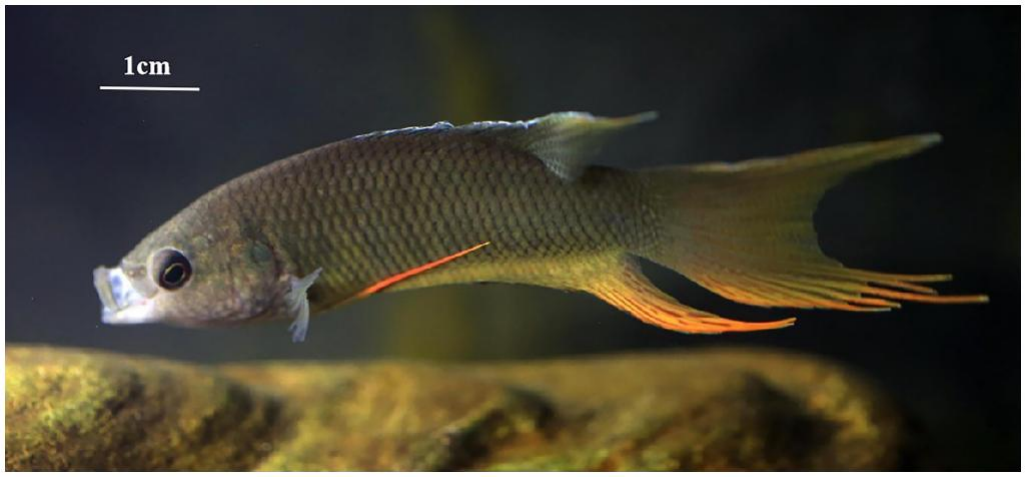
Image of *M. hongkongensis* from the Hainan Wanquan River, taken by Shen Zhixin.

Specimens of adult *M. hongkongensis* (Figure 1) were collected from the Wanquan River, Hainan province (19°11′49.36″N, 110°47′60.59 E) and deposited in the Hainan Academy of Ocean and Fisheries (http://www.hnhky.cn/, Qingfeng Zhang, zhangqf@hnhky.cn) under the voucher number HNFF0930101. Total DNA was extracted using an Ezup DNA kit and sequenced using Illumina 6000. The genome was assembled using the GetOrganelle (Jin et al. 2020). The annotated sequence was submitted to GenBank with the accession number OQ630878.1, and the read coverage depth map is shown in Figure S1 (Supplementary material). A phylogenetic tree was constructed by software RAxML (Stamatakis 2014), using sequences from Genbank: *M. hongkongensis* (MN128300.1), *M. erythropterus* (NC_029946.1), *M. opercularis* (NC_025932.1), *Betta apollon* (LC433688.1), *Trichopodus pectoralis* (KY606170.1), *Trichogaster trichopterus* (KP100265.1), *Colisa fasciata* (KP301136.1), *Colisa lalia* (AP006039.1), (Liu et al. 2019); *Betta splendens* (AB571120.1), (Yu et al. 2016); *Epinephelus hexagonatus* (NC_065829.1), (Wang et al. 2022); *Alces alces* (JN632595.1), (Hassanin et al. 2012); *Centropyge eibli* (KT001113.1) (Shen et al. 2016).

## Results

**Figure 2.**
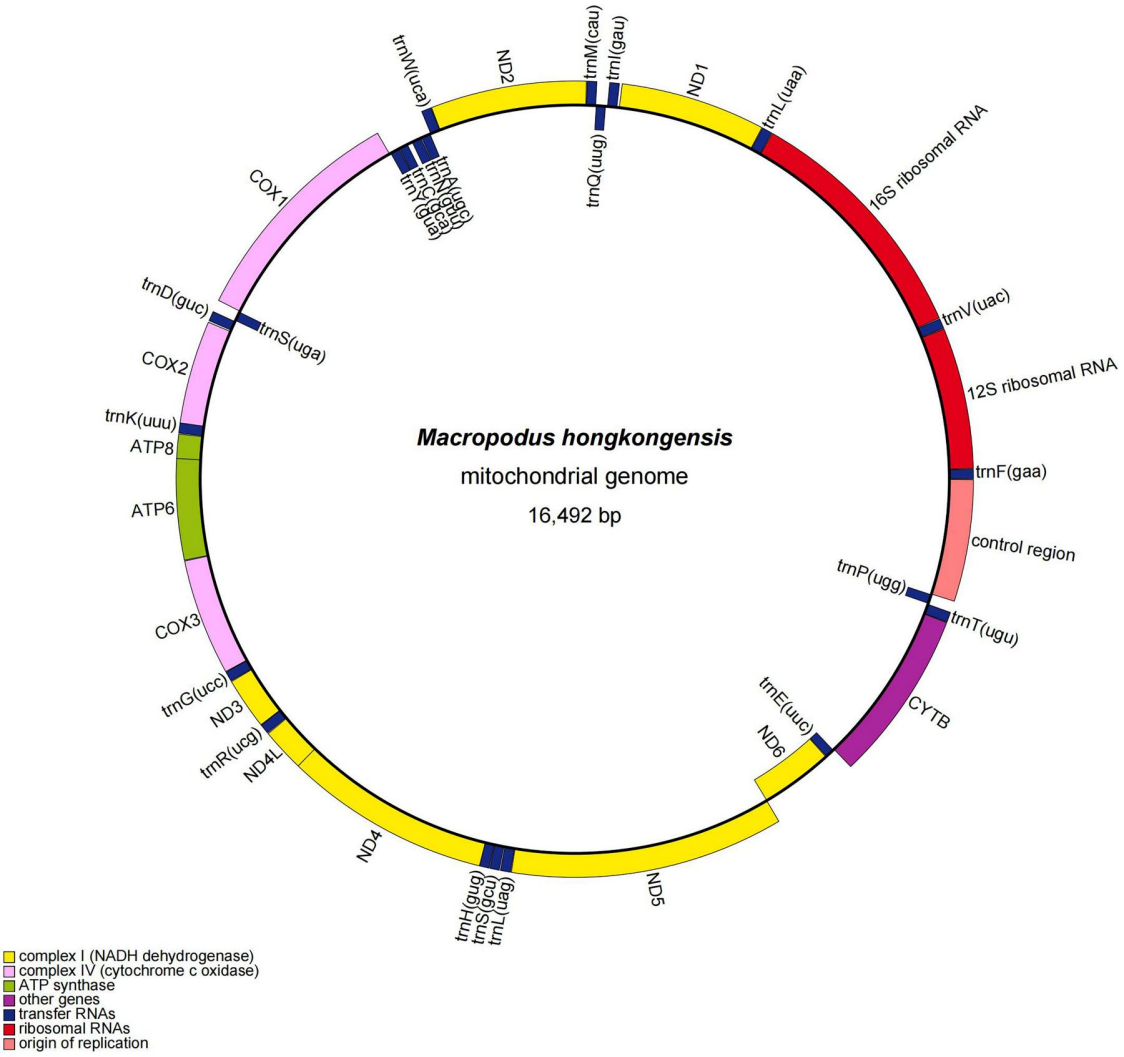
Mitogenome gene map of the *M. hongkongensis* (OQ630878.1).

The complete circular mitochondrial genome of *M. hongkongensis* of the new population was 16,492 bp in length (Figure 2) with a base composition of 30.30, 24.90, 29.80, and 15.00% of A, C, T, and G, respectively. The composition showed a slight bias toward A + T content (60.10%), which was consistent with other Macropodus fishes (Xu et al. 2016). The mitogenome contained 13, 22, and 2; protein (PCGs), tRNA and rRNA-coding genes, respectively, along with a control region. All PCGs had an ATG start codon, except COX1 (GTG) and ND6 (CTA). Four PCGs (ND1, ATP6, ATP8, and ND5) terminated with TAA, and eight PCGs (ND2, COX2, COX3, ND3, ND4L, ND4, ND6, and CYTB) ended with the incomplete stop codon TA– or T––. Except for the ND6 and the eight tRNA genes, all other genes were encoded on the heavy strand (H).

## Conclusion

**Figure 3.**
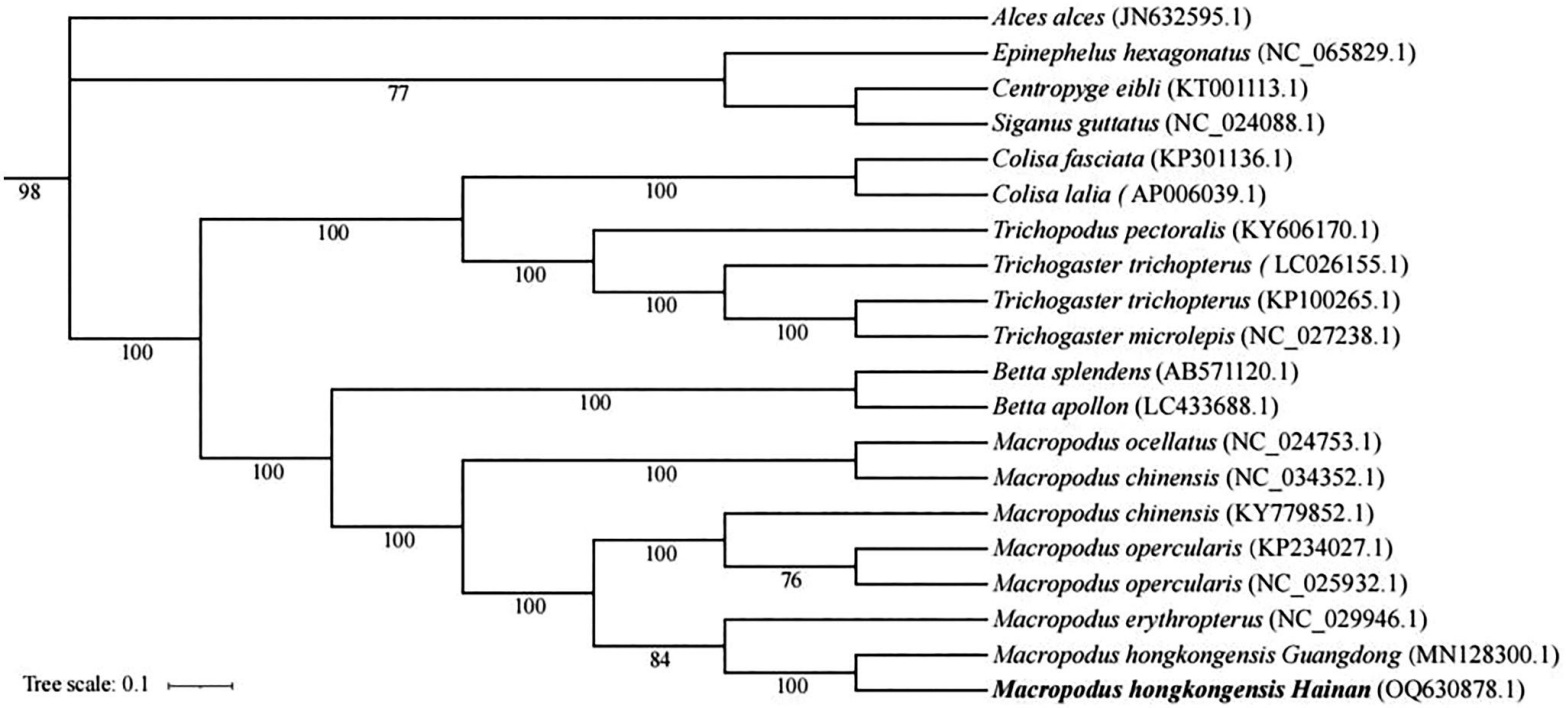
Phylogenetic tree of *M. hongkongensis* inferred by ML. The position of the Hainan population is shown in bold.

In the phylogenetic analysis, *M. hongkongensis* of the Hainan population was most closely related to the Guangdong population (Figure 3) and the nucleotide sequence divergence of 13 protein coding genes between the two population was 4.0%, smaller than the interspecific distance of *Macropodus erythropterus* (13.9%). These results offer molecular markers and a reference for conservation research of *M. hongkongensis* in different populations.

## Supplementary Material

Supplemental Material

## Data Availability

The genome sequence data that support the findings of this study are openly available in GenBank at [https://www.ncbi.nlm.nih.gov] under the accession number: OQ630878.1. The associated **BioProject**, **SRA**, and **Bio-Sample** numbers are PRJNA955001, SRR24305076, and SAMN34163124, respectively.
