# Supplementary figures and images for: The complete mitochondrial genome of a new geographical population of freshwater fish Macropodus hongkongensis (Freyhof & Herder, 2002)
Source: Mitochondrial DNA B Resour. 2023 Nov 23;8(11):1298–300. doi: 10.1080/23802359.2023.2278819 (PMC10769546; doi:10.1080/23802359.2023.2278819)

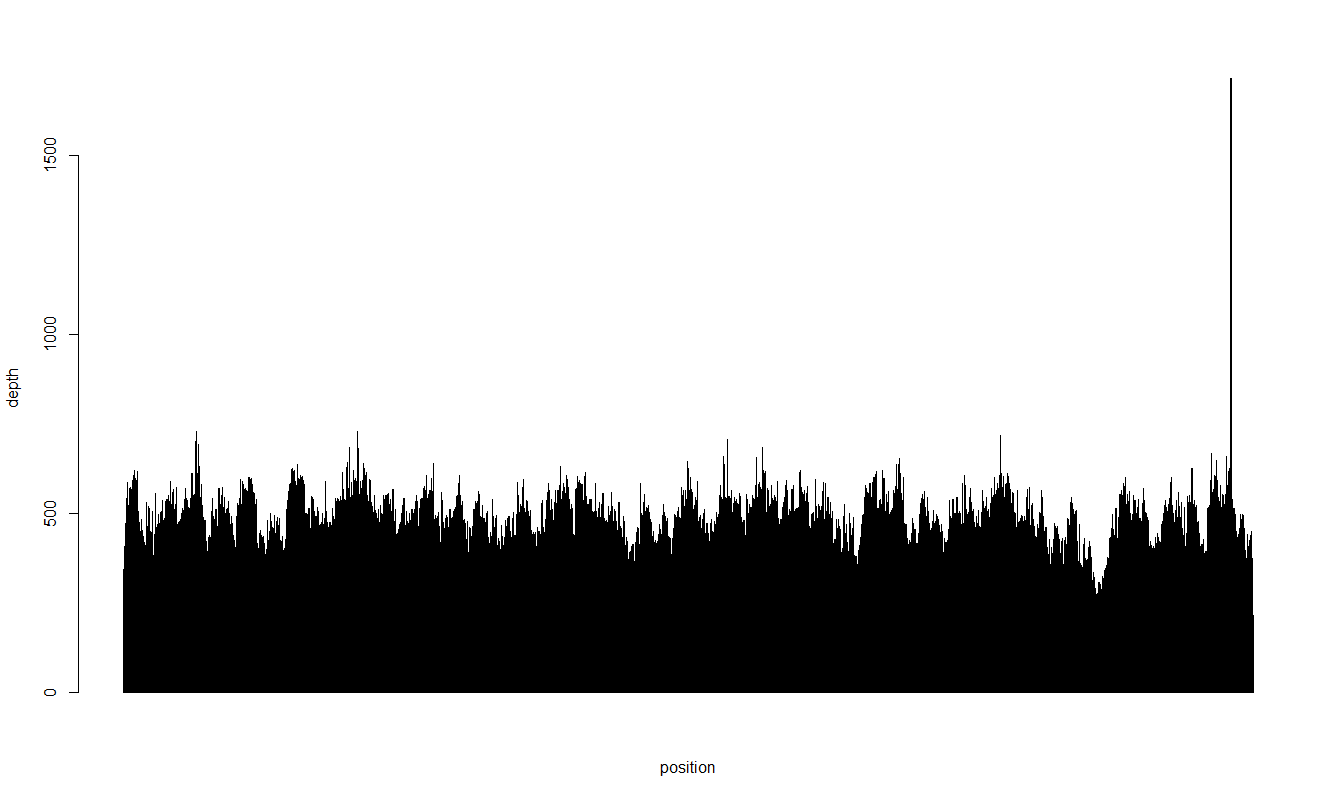

Supplement: Supplemental Material [file TMDN_A_2278819_SM0112.tiff]
